# Health Concerns of Various Nanoparticles: A Review of Their in Vitro and in Vivo Toxicity

**DOI:** 10.3390/nano8090634

**Published:** 2018-08-21

**Authors:** Marziyeh Ajdary, Mohammad Amin Moosavi, Marveh Rahmati, Mojtaba Falahati, Mohammad Mahboubi, Ali Mandegary, Saranaz Jangjoo, Reza Mohammadinejad, Rajender S. Varma

**Affiliations:** 1 Cellular and Molecular Research Center, Iran University of Medical Sciences, Tehran P.O. Box 1449614525, Iran; Maa.biology92@gmail.com; 2 Department of Molecular Medicine, National Institute of Genetic Engineering and Biotechnology, Tehran P.O Box 14965/161, Iran; a-moosavi@nigeb.ac.ir; 3 Cancer Biology Research Center, Cancer Institute of Iran, Tehran University of Medical Sciences, Tehran P.O. Box 13145-158, Iran; m_rahmati@sina.tums.ac.ir; 4 Department of Nanotechnology, Faculty of Advance Science and Technology, Pharmaceutical Sciences Branches, Islamic Azad University of Tehran, Tehran P.O. Box 1916893813, Iran; Falahati@ibb.ut.ac.ir; 5 Department of Midwifery and Reproductive Health, Faculty of Nursing and Midwifery, Abadan School of Medical Sciences, Abadan P.O. Box 517, Iran; Mm59m@yahoo.com; 6 Pharmaceutics Research Center, Institute of Neuropharmacology, Kerman University of Medical Sciences, Kerman P.O. Box 1355576169, Iran; 7 Neuroscience Research Center, Institute of Neuropharmacology, and Department of Pharmacology & Toxicology, School of Pharmacy, Kerman University of Medical Sciences, Kerman P.O. Box 7616911319, Iran; alimandegary@yahoo.com; 8 School of Medicine, International Branch, Shiraz University of Medical Sciences, Shiraz 7134845794, Iran; 9 Regional Centre of Advanced Technologies and Materials, Faculty of Science, Palacky University in Olomouc, Šlechtitelů 27, 783 71 Olomouc, Czech Republic

**Keywords:** nanoparticles, toxicological effects, organ-specific effects

## Abstract

Nanoparticles (NPs) are currently used in diagnosis and treatment of many human diseases, including autoimmune diseases and cancer. However, cytotoxic effects of NPs on normal cells and living organs is a severe limiting factor that hinders their use in clinic. In addition, diversity of NPs and their physico-chemical properties, including particle size, shape, surface area, dispersity and protein corona effects are considered as key factors that have a crucial impact on their safe or toxicological behaviors. Current studies on toxic effects of NPs are aimed to identify the targets and mechanisms of their side effects, with a focus on elucidating the patterns of NP transport, accumulation, degradation, and elimination, in both in vitro and in vitro models. NPs can enter the body through inhalation, skin and digestive routes. Consequently, there is a need for reliable information about effects of NPs on various organs in order to reveal their efficacy and impact on health. This review covers the existing knowledge base on the subject that hopefully prepares us better to address these challenges.

## 1. Introduction

**Figure 1:**
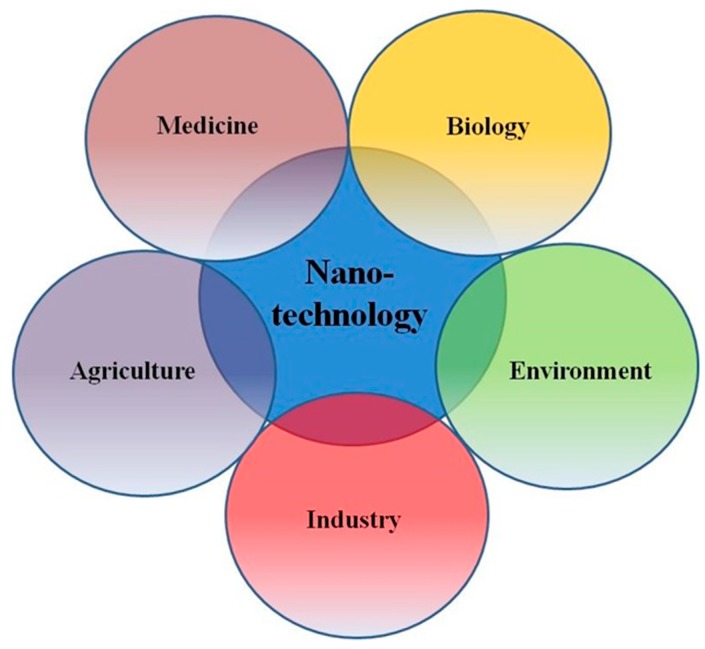
Nanotechnology transformative innovations in medicine, agriculture, industry, environment, and basic biological sciences.

Nanoparticles (NPs) have become widely used in electronics, agriculture, textile production, medicine, and many other industries and sciences (Figure 1) The International Organization for Standardization define NPs as structures whose sizes in one, two, or three dimensions are within the range from 1 to 100 nm [1,2,3,4,5]. Apart from size, NPs may be classified in terms of their physical parameters, e.g., electrical charge; chemical characteristics, such as the composition of the NP core or shell; shape (tubes, films, rods, etc.); and origin: natural NPs (NPs contained in volcanic dust, viral particles, etc.) and artificial NPs, which are the focus of this review [6]. NP toxicity for living organisms, however, is the main factor limiting their use in treatment and diagnosis of diseases. At present, researchers often face the problem and side effects related to their toxicity. In this respect, the choice of an adequate experimental model for estimating toxicity in vitro (cell lines) and in vivo (experimental animals) ones is of paramount importance. NPs can enter into the body through inhalation, skin, and digestion, depending on their physicochemical characteristics and mode of their production [7]. The interactive contact with the body, depending on the type of compounds in NPs, can be respiratory, digestive, or through skin or blood [8]. Some of NPs, such as ZnO and TiO_2,_ have the ability to block UV rays and are extensively used in various health products on the market, which raises concerns about their risks to health, safety and the environment as they are dispersed in the environment. According to primary studies, NPs can enter human body in different ways and they can access vital organs in the body through the blood flow and induce damage to tissues and cells [1,7,8]. Although the mechanism of NPs in this regard is not truly established, researchers have associated the toxicity of NPs to parameters such as particle shape, size, dispersity, surface charge and protein corona effects. Several studies have indicated that NPs activate oxidative stress and expression of genes involved in inflammation [9,10,11]. NPs can enter the human body through respiration, ingestion, and injection and consequently accumulate into different tissues and organs [11,12,13,14]. NPs can even reach the brain by breaking the strong connection between cells and passing through the blood–brain barrier (BBB); they attach to the cells containing CXCR6 chemokine receptor and overcome tight injunction in the BBB [15]. The NPs’ passage through the membrane, their performance, and their cell metabolism are still being studied and discussed. Thus, herein, we attempt to explain a part of the NPs performance that hopefully can answer whether NPs have destructive and toxic effects on organs, or are they safe enough [6]. Development of safe, biocompatible NPs that can be used for the diagnosis and treatment of human diseases can only be based on a complete understanding of the interactions between all of the factors and mechanisms underlying NP toxicity (Figure 1).

Although the safety of many of their chemical components in medicine has been examined, the toxic effect of NPs may be caused by their unique physical and chemical properties, which define the specific mechanisms of interaction with living organs, tissues, and cells. In general, this rationalizes the importance of studying the causes and mechanisms of the potential toxic effect of NPs.

## 2. Medical Applications of Nanoparticles

In medicine, NPs can be used for diagnostic or therapeutic purposes. In diagnosis, they can serve as fluorescent labels for detection of biomolecules and pathogens and as contrast agents in magnetic resonance and other studies. In addition, NPs can be used for targeted delivery of drugs, including protein and polynucleotide substances; in photodynamic therapy and thermal destruction of tumors, and in prosthetic repair [16]. Some types of NPs have been used extensively in drug delivery, diagnosis of diseases and the provision of biologic sensors; several nanometals have been produced and evaluated, but gold and silver are the most widely used. These particles can be prepared in different sizes and shapes, with a small particle size distribution. One of the unique features of these particles is their optical behavior change by changing the particle size, meaning that NPs of different sizes exhibit different colors at visible wavelengths. This feature can be used for diagnosis of the disease and eventual drug delivery to facilitate both these processes. The surface variation of these particles is easy to manipulate as various ligands such as sugars, peptides, proteins, and DNA can bind to these particles [17]. 

Iron oxide superparamagnetic NPs are an important and widely used category of inorganic materials used in drug delivery that can be prepared by chemical procedures such as co-precipitation method or via biological means with the help of bacteria. Easy modification of the particles’ surface, as well as direct bonding of the ligand to them, are salient features of these compounds. In addition, having superparamagnetic property enables the use of these compounds in targeted drug delivery via the magnetic field. Magnetic NPs loaded with a drug can be guided to a specific place in the body by the application of an external magnetic field, thereby bringing the drug to a specific place. For example, Fe_3_O_4_ (magnetite), γ-Fe_2_O_3_ (maghemite, ferrimagnetic) and superparamagnetic iron oxide NPs (SPIONs) are the major NPs used in drug delivery. These particles are typically coated with polymers such as dextran or chitosan to enhance their biocompatibility [18]. Two classes of compounds that have recently been highly emphasized in the drug delivery are carbon nanotubes and fullerenes (also known as Buckyballs); their size, shape and surface properties have empowered their use in drug delivery. Single-wall carbon nanotubes and C60 fullerenes have a diameter of about 1 nanometer, which is half the diameter of a DNA helix. Because of their small size, these particles can easily pass through the membranes and biological barriers and penetrate into the cell. These structures allow for surface engineering with their high surface to volume ratio. The surface of these particles can be coated with various compounds to enhance solubility and biocompatibility, as well as the delivery of different materials including biological molecules such as proteins, DNA and drugs. Pharmaceutical compounds are often loaded onto or inside these structures. Targeting and simultaneous transfer of two or more compounds are additional interesting features of importance in drug delivery by these particles [17]. 

The term liposome was coined in 1961 by Alec D. Bangham. These double-layer vesicles consist of a liquid part enclosed in a double layer lipid membrane, which is often a natural or synthetic phospholipid. Amphiphilic nature, biocompatibility and the ease of surface changes are among the factors that initiated the use of these structures as an option for drug delivery [17,19]. Another example of lipid nanostructures is solid lipid NPs (SLNs) that form a solid lipid matrix consisting of triglycerides, lipids, fatty acids, steroids and waxes, and have a size less than 1 μm. In order to increase the stability of these particles, surfactant compounds are often deployed in their formulation. These NPs can be used to load and carry drugs with very low solubility in an aqueous medium, release them in a specific time frame, and transfer them to the desired site via, for instance, oral methods or injection [20]. Another very commonly used materials, in the form of NPs for drug delivery, are polymers, natural or synthetic, which need to be biocompatible, non-toxic and free from leachable impurities besides comprising an appropriate physical structure and a desired half-life. Polymer NPs are often selected from biodegradable types, the main advantage being their high stability and their scale-up production in large quantities. These involve a large number of compounds that form vesicular systems (nanocapsules) and matrix systems (nanospheres); the drug is kept inside a polymeric cavity in nanocapsules, while it is dispersed in a polymer matrix in nanospheres [17,20]. Polymer micelles are self-assemblies of macromolecules that consist of block copolymers with non-covalent bonds; block copolymer micelles have a core-shell structure. Specific properties of the micelles, such as critical micellization concentration (CMC), aggregation number, size and shape of their final structure depend on the structure and length of the polymer chains in the copolymer block. Polymer micelles usually have a low CMC, which affects their ability to increase the solubility of loaded drugs and the resistance of micelles [20,21] which can be effective in reducing the speed of drug release. These structures also have more mechanical and biological stability compared to liposomes because the interaction of vesicles and macrophages is less common in these structures, resulting in more protection for the drug. Despite all these advantages, there is still no formulation for this structure class in the pharmaceutical market. Hydrogel NPs are three-dimensional polymer structures used to encapsulate and transfer drugs. These structures swell in water or in the bioenvironment and carry a large amount of fluids inside. There are also stimulus-responsive hydrogels which release the drug under specific environmental changes, such as temperature and pH changes. These systems have been used to transfer DNA and proteins, heal wounds, make biosensors, and engineer tissues [17].

## 3. Mechanisms of Nanoparticle Toxicity: NP-Cell Interactions

**Figure 2:**
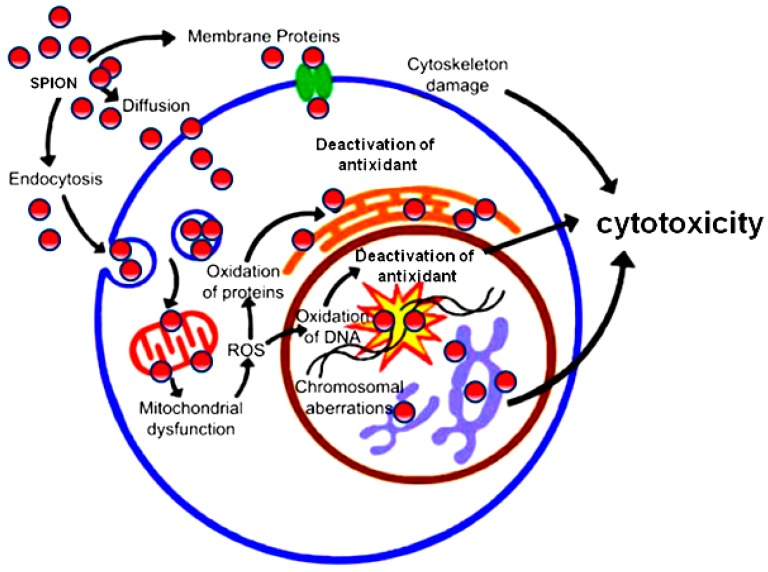
The main routes of nanoparticles (NP) entry into the cells and their subsequent intracellular mechanism(s) [24].

Surface properties of NPs, namely hydrophobicity and hydrophilicity, affect many of the biological environmental responses of these structures, such as interaction with plasma proteins, cellular uptake and phagocytosis, stimulation of the immune system and particle removal. The surface properties of nanoparticles result in different cellular responses such as adhesion, growth and differentiation. The oxidative stress is induced by NPs through physicochemical interaction in the cell membrane as they generate ions which cause toxicity in the cell membrane surface and that can be exploited to eliminate cancer cell [22]. The higher the diameter of the NPs, the more their interaction with the surface of the cell membrane and the higher the level of cellular toxicity. The cell membrane is complex and dynamic comprising proteins and extracellular polymeric materials. As shown in Figure 2, the penetration of NPs occurs through intrusion at the diffusion, endocytosis and membrane proteins such as phospholipid layer. NPs are subsequently localized in endosomes and nucleus, degraded in lysosomes or recycled back to the plasma membrane although the mechanism may still be unclear. The toxicity of Au NPs with a diameter under 100 nm have been explored. In the range of 3, 5, 50 and 100 nm, the toxicity was observed for the biggest and smallest sizes which included apoptosis, oxidative stress, organelles and DNA destruction, and mutagenesis [23]. NPs enter cell through endocytosis and their toxicity is predominantly through an increase of reactive oxygen species (ROS) levels in the cell (Figure 2).

**Figure 3:**
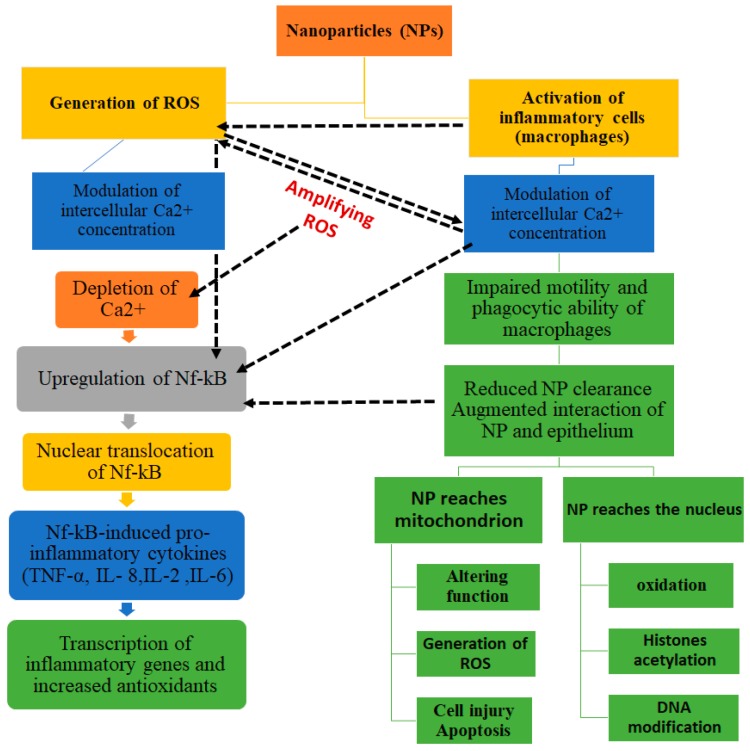
The most common mechanisms underlying NP cytotoxicity.

NPs can also increase inflammatory factors such as TNF-α, ll-8, ll-6, ll-1, and ultimately cause mitochondrial damage (Figure 3) [24,25,26]. The interaction of NPs with the cell surface ligand and membrane receptors is the main connection route for drug delivery and this is implemented through endocytosis. Recently, with the aim of reducing the toxicity of NPs in drug delivery, amphipathic Au NPs have been used. Being hydrophobic, they are protected against microbial attacks, swelling or changes in pores due to pH changes as these NPs pass through membrane without damage; a behavior reminiscent of the cyclic citrullinated peptide (CCP) for Rheumatoid arthritis therapy. α-helix protein has a hydrophilic part and a hydrophobic part and CCP bonds with cationic group, enters the cell and connects with the negative charge remained from the membrane [27]. The factors that are important in the connection of NPs to the cell surface protein are surface charge and hydrophobicity of the particles and the particles reaction with the protein tail or phospholipid head; the cationic level being stronger than the anionic level in this process. The interaction of NPs with water molecules, their hydrophobic property, is in fact a factor for drug delivery properties for medications whose transfer is otherwise difficult. Coating NPs with ligands impacts the size, ligand density, receptor emission, and free energy changes. The rod and cylindrical shapes of NPs, compared with the spherical shape, need more time for wrapping and this is due to the thermodynamic force for engulfment [28]. The interaction of NPs with macromolecules such as protein has been explored and such interface can result in structural changes of proteins [29]; proteins have multiple 3D structures and some structures change after attachment of NPs due to diversity of amino acids and the protein performance. NPs such as C60 fullerenes and SWCNTs with attachments, for destruction of the activity of enzymes such as human immunodeficiency virus type 1 protease (HIV-1p) and S-DNA-glutathione, are used for therapeutic purposes [30]. But these features may also underline their toxicity in living organ, the key mechanism responsible for the cytotoxic effects of NPs being oxidative stress that results in an intracellular disharmony and consequently the increase of ROS (Figure 2). DNA strand damage is via base changes namely hydroxy deoxyguanosine formation and, when DNA is not repaired, the cell cross-linking results in the occurrence and progression of cancer. Oxidative stress subsequently activates various signaling pathways that may lead to cell death [31].

Briefly, the most common mechanisms of NP cytotoxicity entail the following (Figure 2 and Figure 3):

1. NPs may cause oxidation via increase of reactive oxygen species (ROS)
2. NPs may damage cell membranes by perforating them
3. NPs damage components of the cytoskeleton, disturbing intracellular transport and cell division
4. NPs disturb transcription and damage DNA, thus accelerating mutagenesis
5. NPs damage mitochondria and disturb their metabolism, which leads to cell energy imbalance
6. NPs interfere with the formation of lysosomes, thereby hampering autophagy and degradation of macromolecules and triggering the apoptosis
7. NPs cause structural changes in membrane proteins and disturb the transport of substances into and out of cells, including intercellular transport
8. NPs activate the synthesis of inflammatory mediators by disturbing the normal mechanisms of cell metabolism, as well as tissue and organ metabolism (Figure 3).

The penetration of NPs can occur through diffusion, endocytosis and membrane receptor proteins. NPs are then localized in late endosomes, mitochondria, endoplasmic reticulum (ER) or nucleus, then induce signaling pathways that are mostly depended on ROS. Mitochondrial ROS can lead to accumulation of more levels of ROS and resultant oxidative stress may disrupt protein folding process, causing ER stress and induce DNA damage, leading to activation of cell death pathways [32].

Although some NPs, such as Ag NPs, are used as an antimicrobial agent because of this mechanism, inappropriate use of these NPs can damage other cells instead of microbes. For example, Ag NPs can be used to disinfect wounds and prevent the growth of bacteria in that area. They can prevent bacterial growth and replication through the above mechanisms and heal the wound. But, it should be noted that the same NPs can also affect the cells of human body around the injury site and cause cell death. 

### 3.1. The Effect of NP on the Protein Conformational Changes

A number of techniques such as nuclear magnetic resonance (NMR) spectroscopy [33], X-ray crystallography [34], circular dichroism spectroscopy [35], isothermal calorimetry [36], differential scanning calorimetry [37], fluorescence spectroscopy [38], and UV-visible spectroscopy [39] have been widely used for analyzing the protein-NP interactions. The NP-induced conformational changes and subsequent corona formation depends on several factors such as, protein type, NP type, size of NP, shape of NP, pH and the temperature.

**Figure 4:**
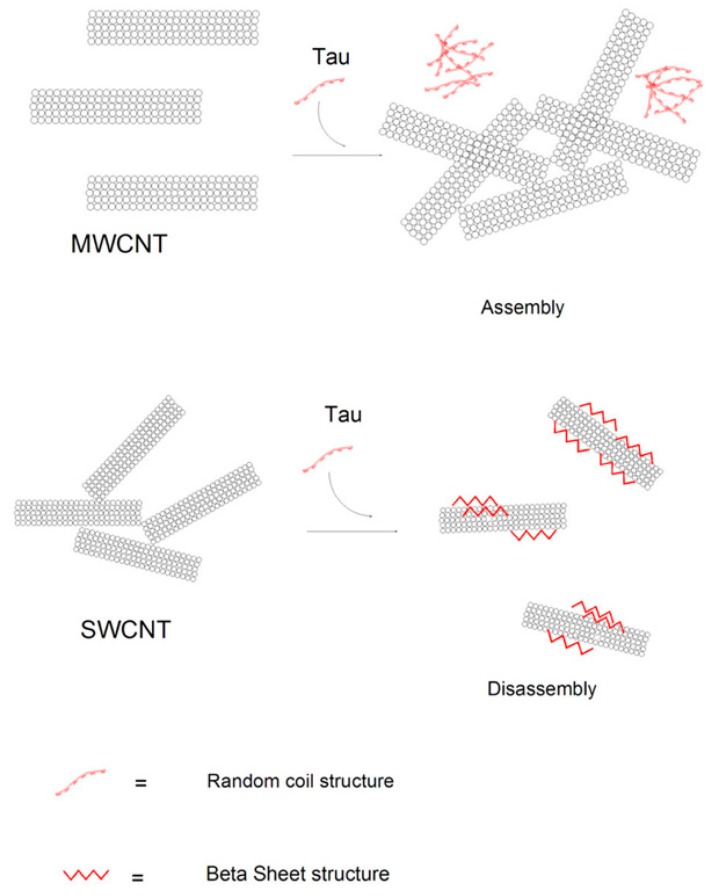
Schematic illustrating single-walled carbon nanotubes (SWCNTs)-induced interactions with tau protein structure, resulting in pronounced conformational changes and corresponding denaturation compared to multi-walled carbon nanotubes (MWCNT) [40].

Subtle changes in the structure of NPs affect their surface properties and subsequent interaction with proteins. The interaction of the single wall carbon nanotube (SWCNT) and multiwall carbon nanotube (MWCNT) of varying diameter with tau protein was investigated by different methods [40]. The circular dichroism bands of the tau protein after concentration variation of SWCNT showed a remarkable increase of β-sheet content indicating that the binding of tau with SWCNT causes the protein folding and more compact structure of natively unfolded structure of tau protein. Also, as shown in Figure 4, the binding of MWCNT has not altered the secondary structure of tau protein and has resulted in the protein aggregation. This study showed that SWCNT induced stronger interactions with tau protein, causing more pronounced structural changes [40]. Also, transmission electron microscopy (TEM) observation showed that tau protein can bind to the surface of SWCNT thus dispersing it, whereas tau protein cannot attach on the MWCNT surface and eventually ends up in MWCNT agglomeration [40]. Surface functionalization of NPs can also influence the protein adsorption and subsequent NP induced conformational changes. Protein surface residues form an interaction with energetically favorite counterparts on the NP surface based on their charge, hydrophobicity, and hydrophilicity [41]. Thermodynamic parameters can stipulate the kind of interaction between protein and NPs namely standard enthalpy change (ΔH^0^), standard entropy change (ΔH^0^), standard entropy change (ΔS^0^), and standard Gibbs free energy change (ΔG^0^). When ΔH^0^ and ΔS^0^ are negative, then the main interacting forces between the NP and protein are hydrogen bonds and van der Waals interactions. However, if ΔH^0^ is almost zero and ΔS^0^ is positive, then the common involving bonds between NP and protein are electrostatic interactions [42].

### 3.2. The Effect of Protein Corona on the Toxicity of NPs

After injection of NPs into the bloodstream, there is a competition between different biological molecules to interact with the surface of NPs (Vermann effect). In the first step, the smallest abundant proteins are adsorbed onto the surface of the NPs, however, over time, they are replaced by proteins with higher affinity [43]. The structure and composition of the protein corona depends on the physicochemical properties of the NPs, the physiological environment and the duration of exposure in that environment. Protein corona changes the size and surface composition of nanomaterials and provides them a new biological identity which determines the physiological responses including aggregation, cellular absorption, and the half-life of NPs in the blood, signaling synthesis, transfer, accumulation and toxicity. The corona on NPs is complex with no general protein corona specific to NPs [44]. Albumin, immunoglobulin G (IgG), fibrinogen, and Apo lipoproteins are found in the corona of all studied NPs; these proteins are prevalent in the blood plasma and hence, over time, may be replaced by proteins with lower concentration but higher affinity on the surface of NPs. Molecules that are weakly attached to the NP and interact with it are soft coronas. NPs with a pre-formed agent group, such as polyethylene glycolated (PEGylated) NPs, contain only one weak covering corona and no hard corona [45]. Protein corona reduces the toxicity of NPs by reducing their cellular absorption. In other words, NPs with less protein corona have more cellular absorption and are thus more cytotoxic. This phenomenon has been reported for CNTs [46], graphene oxide Nano sheets [47] and biopolymer NPs in various cell environments [48]. In the case of common toxic nanomaterials, such as positively charge polystyrene NPs, protein corona has a protective role against membrane damage [49,50]. 

### 3.3. The Effect of Protein Corona on Non-specific Cellular Uptake

The specific entry of NPs into the cell is accomplished by a receptor-specific ligand. Non-specific cellular uptake is a random process of the cell performed without bio molecular control. The amount of NP entry into the cell depends on protein corona. The non-specific cellular uptake of oligonucleotide-mediated AuNPs has been investigated which showed that their absorption significantly increased in an environment free of serum proteins [51]. Similarly, the cellular absorption of FePt NPs with quantum dots (QDs) is reduced dramatically in HeLa cells through the formation of protein corona [51].

### 3.4. The Effect of Protein Corona on Bio-distribution of NPs

The nature of the NP’s core, whether non-polymeric or polymeric, shows that pre-coating increases NP’s persistence in the blood and reduces the clearance rate. A study disclosed that the life of bovine serum albumin (BSA)-coated Nano drugs was 6 times more than that of non-coated ones [48].

### 3.5. The Effect of Surface Charge of NPs on Their Toxicity

NP hydrophobicity and surface charge changes the biological distribution of NPs due to their effect on the level of interactions between NPs and the immune system, plasma proteins, extracellular matrix, and non-target cells. Hydrophobic/charged NPs are less persistent in the circulation due to the opsonization of particles by plasma proteins and ultimately by the RES system. Positively charged NPs are attached to negatively charged non-target cells in a non-specific manner; hydrophobic groups on the NP surface induce NP aggregation, which accelerates the identification and relocation by the respiratory (RES) system. In order to reduce this interaction, the surface of the particle is covered with hydrophilic PEG, which reduces the level of opsonization and hence increases particles’ persistence in the circulation [52].

## 4. The Effects of Physicochemical Properties of NPs on Cytotoxicity

In fact, a unique property of nanomaterials is their high surface-to-volume ratio which endow them with useful characteristics, but is ironically that trait is also associated with unique mechanisms of toxicity. Toxicity has generally been thought to originate from nanomaterials’ size and surface area, composition, shape, and so forth as discussed in the following sections.

### 4.1. The Effect of NPs Size on Cytotoxicity

NP cytotoxicity is affected by changes in NP size [53] and is dependent on the surface-to-volume ratio [54]. Sedimentation velocity, mass diffusivity, attachment efficiency, and deposition velocity depend on the size of the NPs [55]. The size of NPs plays an important role in interacting with the biological system, and it has been revealed that various biological mechanisms such as endocytosis, cellular uptake, and particle processing efficiency in the endocytic path depend on the size of materials [56]. NP size affects the ion release rate, the smaller the size, the faster the release rate and the more the interaction with cell membrane; therefore, it will penetrate into the cell and induces higher toxic effect [57]. In general, size-dependent toxicity of NPs can be related to their ability to enter biological systems.

NP sizes of less than 50 nm administered through intravenous injection reach the tissues faster than 100–200 nm NPs and exert stronger toxic effects. If the size of NPs is reduced, their contact surface will increase and the level of oxidation and DNA damage will also rise. The size of NPs indicates their pharmaceutical behavior, that is, sizes of less than 50 nm quickly connect to all tissues and exert toxic effects. NPs larger than 50 nm are used by the RES, which stops its path to other tissues. But again, organs like the liver and spleen are the main targets of oxidative stress.

The size of NPs has a direct effect on their physiological activity. NPs of size less than 1 μm enter the cell and their effects are unknown; those larger than 1 μm do not easily enter the cell, but they replace a series of proteins that are absorbed at their surface and react with the cell. Accordingly, the NPs size is effective in cell endocytosis [58]. For example, Kim et al. showed that the toxicity of Ag NPs in in vitro model on MC3T3-E1 and PC12 cells is size-dependent. NPs size and dosage affected cell viability as it produced intracellular ROS, LDH release is a useful method for detection of necrosis [59].

### 4.2. The effect of NPs Structure and Shape on Cytotoxicity 

NPs come in a variety of shapes, such as spherical, rod-like, filament, and plate-shaped which influences their toxicity [60]. 

The shape of NPs is effective in the membrane packaging process in endocytosis and phagocytosis [61]; endocytosis of spherical NPs is faster than tubular NPs [62]. Non-spherical NPs are more exposed to blood flow and have more toxic effects.

CNTs can be of single-walled CNTs (SWCNTs) or multi-walled (MWCNTs) class that affect their mechanisms on cell viability; SWCNTs produce more ROSs that MWCNTs [63]. The toxicity of Nano-carbons was found to be dependent on shape and concentration [64]. TiO_2_ NPs cause oxidative damage to DNA, induce lipid peroxidation and micronuclei formation in the presence of light, and these NP-induced effects change with shape [65].

### 4.3. The Effect of NPs Surface on Cytotoxicity

Surface charge of NPs affects biological aspects such as absorption, colloidal behavior, plasma protein binding, and passage through the blood-brain barrier [66]. Negatively charged NPs have more cellular absorption than the positive and neutral NPs due to resistance by plasma proteins, which causes hemolysis and platelet aggregation and eventually toxicity.

NPs surface affects absorption level of ions and biomolecules that may alter cellular response. In addition, surface charge determines the colloid behavior which is the response of the organism to changes in NPs shape and size in the form of cellular accumulation. The effect of surface chemistry of NPs on human immune cells and RBCs in in vivo and in vitro models has been investigated [67]. For instance, the effect of silicon surface charge on cell lines reduced the ATP and genotoxicity for negative hydrophilic and hydrophobic charge relative to hydrophilic, positively charged amine-modified surfaces. The interaction between NPs and cells initially depends on the nature of NPs surface. The incubation of NPs with cells may interfere with cell adhesion, affecting cellular properties such as morphology, cytoskeleton, proliferation, and even survival. Of course, it is worth noting that the surface of NPs and the groups on their surface have a significant effect on adhesion. For example, bare iron oxide NPs with an approximate diameter of 50 nm have 64% less cell adhesion compared to polyethylene glycol (PEG) coated ones. This can be due to the difference in the interaction of NPs/cells with different charges in the presence or absence of surface-coating agents, while the metabolism of the nanotube function is different [68].

### 4.4. The Effect of NPs Concentration on Cytotoxicity

The 2 mg/mL concentration of silicon had a toxic effect on the cell, but no toxic effect was observed in 4 mg/mL [69]. Varied concentrations of Ag NPs altered mitochondrial function and LDH release; the toxicity changed with changing concentrations, however [9]. 

## 5. In Vivo Study of Nanoparticle Toxicity

In addition to the numerous study of the behavior of NPs in the in vivo model is being extensively studied. These studies are focused on the biomedical applications of NPs, the NPs toxicity for living organisms remains an important topic. Although NPs are highly promising for difference medical applications, they are potentially side effect. This side effects cannot be estimated exactly in vitro, following from the comparison of the in vivo and in vitro effects of NPs. Metal oxide NPs such as titanium dioxide (TiO_2_) are among the most used NPs, in particular, in environment protection measures. Therefore, it was important to evaluate their toxicity in the bioavailability, in experiments with their injection to experimental animals. This study has been performed by Kiss B et al [70]. Experimental animals (rats) were injected with a suspension of TiO_2_ NPs at a dose of 15 μg/cm^2^ and their bio distribution, as well as the general condition of the animals, was monitored. The results have shown that the animals have inflammation or another manifestation of a toxic effect observed, within 24 h suggesting that TiO_2_ NPs are relatively hazardous. 

**Figure 5:**
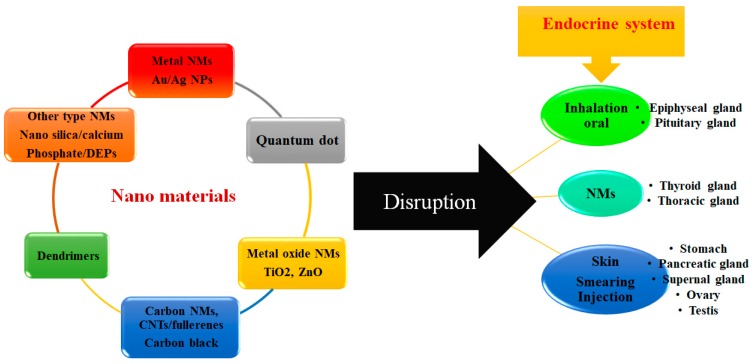
Toxic effects of diverse types of nanoparticles on various organs.

Silver NPs are another example of NPs potentially useful in medicine, because of their antimicrobial activity. Their toxicity and bio distribution were analyzed by Mitra Korani [71] in an experiment where Guiana Pigs were dermal exposure with 100, 1000, 10,000 ppm of silver NPs of different sizes (less than 100 nm). The results have shown a close correlation between dermal exposure and tissue levels of Ag NPs was found and tissue with the following ranking: kidney–muscle–bone–skin–liver–heart–spleen (Figure 5). In histopathological studies, severe proximal convoluted tubule degeneration and distal convoluted tubule were seen in the kidneys of the middle and high-dose animals. Separated lines and marrow space narrow were determined as two major signs of bone toxicities which observed in three different dose levels of Ag NPs. Increased dermal dose of Ag NPs caused cardiocyte deformity, congestion and inflammation. The three different Ag NPs concentration gave comparable results for several endpoints measured in heart, bone and kidney, but differed in tissue concentrations and the extent of histopathological changes. It seems that Ag ions could be detected in different organs after dermal exposure, which has the potential to provide target organ toxicities in a time and dose dependent manner. 

Gold NPs have been shown to be toxic for mice, causing weight loss, decrease in the hematocrit, red blood cell count. In drug delivery using gold NPs, it is also important to know their toxic properties, because the positive effect of their use should overcome over the negative one. Results in one study have been obtained for gelatin NPs modified with polyethylene glycol, which are designed to be used for delivery of ibuprofen sodium salt [72]. The NPs have proved to be nontoxic at the dose that is necessary for effective drug delivery (1 mg/Kg), which has been confirmed by estimation of inflammatory cytokine levels in the in vivo model, as well as histological analysis of their organs. CNTs are among the nano-carbon structures that, due to their hollow and small structure (smaller than red blood cells), play a special role in the field of medicine, such as drug delivery to target cells, bio-sensoring blood glucose, detecting and destroying cancerous cells, tissue engineering, and so on. Recent studies have shown that CNTs can be used for biological purposes, such as crystallization of proteins, and the production of bioreactors and biosensors. The intrinsic fluorescence properties of nanotubes make them suitable biosensors for identifying specific targets in human body tissues, such as cancer tumors. Numerous methods have now been devised to connect DNA molecules and proteins to the internal and external surfaces of nanotubes; this enhances the ability to target and destroy single cancer cells or viral infectious cells [73]. The assembly of special enzymes to nanotubes has resulted in their widespread use as enzymatic biosensors, which allows the identification and measurement of a variety of biological molecules most widely used in the rapid measurement of blood glucose. Recently, the use of CNTs in tissue engineering has attracted the attention of researchers; the key role of CNTs in the culture of tissue cells such as fibroblasts is such an example [73]. 

Quantum dots are among the NPs that are most promising for medical applications. However, they are potentially side effect for health, because they exhibit various toxic effects in both in vitro and in vivo experiments. The primary application of QDs is now in the field of photography and disintegrating biological compounds. Their additional applications include marking single molecules and optical tracking of their behavior. In these methods, QDs act as chemical marks. Biomolecules, such as antibodies, bind to QDs which makes QDs attach, in a purposeful and specific manner, to target molecules or target cells whose surface is covered by supplemented antigens. The binding of antibodies on the surface of QDs to antigens attached to the surface of these specific cells or proteins results in the emission of light from QDs. If there is no target cell or protein in the sample, no emission will be observed. Therefore, optical tracking of cells or biomolecules is possible over an extended period of time. It should be noted that QDs are extensively used in the detection of cancerous tumors. It passes through the BBB pathway and through trigeminal nerve or olfactory epithelium. CdSe/Zn NPs with a diameter of 13 nm have the ability to reach tumor tissue in laboratory mice. Six days after the injection, brain nuclei were isolated and Cd was observed in the brain tissue, but there was no indication of astrocyte damage and nerve inflammation. However, the toxicity of this particle for the nerve tissue needs further investigation. QD toxicity is size-dependent; sizes below 20 nm accumulate in the brain parenchyma. In vitro studies used these NPs to target brain tumors in the cell-line, which in the long term were able to reduce the volume of cancer cells [74]. Similar results were obtained by Zhang et al. [75] where they showed that CdTe QDs predominantly accumulated in the liver, decreasing the amount of antioxidants in it and inducing oxidative stress in liver cells. Cadmium and tellurium ions tend to accumulate in various organs and tissues upon degradation and decay of the cores of CdTe/ZnS QDs. 

## 6. Study of Toxicity in Cell Cultures

Many studies of NP toxicity are carried out in cell cultures serving as models of numerous types of human and animal cell. In some cases, cancer cells are used, specially, for the evaluation of toxic effects of NPs used in cancer chemotherapy. The type of cells is selected according to the potential route by which NPs enter the body. This may be oral uptake (mainly by ingestion), transdermal uptake (through the skin surface), inhalation uptake of NPs contained in the breathing air, or intentional NP injection in clinic. Intestinal epithelium cells are often used in experimental models for studying the toxicity of ingested NPs. In in vitro model, the kinetics of NP uptake by cells and the viability of cells upon the NP uptake are studied. The NPs that use in drug delivery, or those used for imaging, are administered by injection. The toxicity of these NPs is studied in primary epithelial cell cultures. Most commonly, an increase in ROS, GSH, IL-1β, IL-6, IL-8, and TNF-α, are estimated. In addition, various tumor cells (gastrointestinal, human colon, skin, pancreatic PANC-1cells, human lung adenocarcinoma cells, human hepatocellular carcinoma HepG2 cells, human skin carcinoma A431 cells) are used. The toxicity of inhaled NPs is studied using the primary cell lines and different tissues of the respiratory system, including, primary rat brain microvessel endothelial cells (rBMEC), murine neural stem cells (NSCs), human pulmonary cell line (lung adenocarcinoma epithelial A549 cell line), different human epithelial cells and fibroblasts, catla heart cell line (SICH), cardiac microvascular endothelial cells, keratinocyte cell line (HaCaT), human dermal fibroblasts, human immortalized sebaceous gland cell line (SZ95), rat liver derived cell line (BRL 3A), human hepatoblastoma C3A cell line, and embryonic kidney cells (HEK293). The toxicity of the NPs that enter the body trans dermally is usually studied in keratinocytes, fibroblasts, and, more rarely, sebocytes (Table 1).

## 7. Conclusions

Nanoparticles have many biomedical applications due to their unique characteristics such as size, shape, chemistry and charge. However, the signaling pathways through which NPs can produce toxic effects need to be understood better. Recent studies have shown that inflammation, necrosis, ROS and apoptosis are key factors that mediate the mechanism of toxicity of NPs. These results may create a barrier to the use of NPs in diagnosis and in the treatment of diseases for which they are ideally suited. It is important to identify the dose, shape, and the properties of NPs that are responsible for their toxicity in order to reduce their side effect by appropriately modifying the formulation or to use a NP with lower toxicity. The dose of NPs is an important factor in their toxicological profile, along with their accumulation, distribution, metabolism and disposal. In line with this, intravenously injected NPs have a higher toxicity than those administered to the skin. According to the results of various studies, there should be protocols that show which doses and what structures of NPs are more toxic. In general, the problems in the evaluation of NP toxicity are due to the disparity between different toxicological studies performed on the NPs of diverse origins and make-up. Accordingly, the study of NP toxicity in various applications, especially biomedicine applications such as drug delivery, bio-security and NP toxicity, is very crucial. Consequently, there is a need for the development of accepted and specific protocols to identify the actual particle with its surface surroundings and the composition of NPs that renders them toxic. It is hoped that our increased knowledge of NPs lead to their safer design with reduced toxicity so that they can be used for treatment of assorted diseases and drug delivery.

## Figures and Tables

**Table 1:** Toxic effects of nanoparticles on different organs/tissues.

Target	NP	Concentration, (Size), (Time), Route of Administration	Major Outcomes	Cell	In Vitro Effect
Brain	AuNP	0.8–50 μg/mL, (3, 5, 7, 10, 30 and 60 nm), (24 h)	Only the smallest NP tested (3 nm) induced mild signs of cellular toxicity [76].	rBMEC (primary rat brain microvessel endothelial cells)	Non-toxic even at highest concentrations in 24 h [76].
		50 μg/mL (6–120 h)	-	Zebrafish cembryos	Time- and dose-dependent correlating increases in permeability and cytotoxicity of cells [77].
	AgNP	6.25–50 μg/mL, (25, 40 or 80 nm in size), (24 h)	Time- and dose-dependent increase in pro-inflammatory cytokine release and related rises in permeability and cytotoxicity of cells [76].	rBMEC (primary rat brain microvessel endothelial cells)	Time- and dose-dependent increase in pro-inflammatory cytokine release and correlating increases in permeability and cytotoxicity of cells [78].
	Cu	30–50 mg/Kg	increasing toxicity on neuromuscular system and increase NPs penetration of the blood-brain barrier [78].	-	-
	Al	30–50 mg/Kg	increasing toxicity on neuromuscular system and increase NPs penetration of the blood-brain barrier [78].	-	-
	CdSe	1, 10, 20 nm, (24 h)	-	Primary rat hippocampal neuron cells in culture	Decrease of cells viability [79].
	Superparamagnetic iron-oxide nanoparticles (SPION)	208 or 1042 μg/mL of: Ferumoxtran-10; Ferumoxytol (20–50 nm); Ferumoxide (60–185 nm) (3 months)	Increasing uptake NPs into the CNS parenchyma [80].	Murine neural stem cells (NSCs)	Depleted intracellular glutathione levels, altered activities of SOD and GPx, hyperpolarization of the mitochondrial membrane, dissipated cell-membrane potential and increased DNA damage [81].
	TiO_2_	30–45 nm, (2–72 h)	leakage of lactate dehydrogenase (LDH) [82]	Neuro-2A	permeability of NPs in plasma membrane, increasing apoptosis [82].
	ZnO				
	Fe_2_O_3_				
	Al_2_O_3_				
	CrO_3_				
	CNT	PEG-SWCNTs at concentrations of 0.5, 2.1 and 1 mg/mL	Accumulation in the hippocampus which induces oxidative stress [83].	PC12 cells	Decreased mitochondrial membrane potential (MMP), induced ROS and increased the level of lipid peroxide and decreased the activities of superoxide dismutase (SOD), glutathione peroxidase (GSH-Px), catalase (CAT) and glutathione (GSH) [84].
	QD	0.68 mg containing 50 nmol Cd (13.5 nm in size), (6 h) Intraperitoneal	Moderately high quantities of Cd ions was observed in brain tissue but no signs of inflammation or parenchymal damage were detected [74].	Neuron like PC12 cells	Cell death, axonal degeneration [85].
Lung	AgNP	515 g/m^3^, (6 h/day, 5 days/week for 13 weeks), inhalation	Dose- and time-dependent increase in blood Ag nanoparticle concentration was observed along with correlating increases in alveolar inflammation and small granulomatous lesions [86].	-	-
	Cu	0.1–3300 µg/mL, (3 and 24 h)	-	Human pulmonary cell line (lung adenocarcinoma epithelial cell line (A549))	Mitochondria-dependent cellular apoptosis associated with ROS [87].
	Zn				
	CO				
	Sb				
	Ag				
	Ni				
	Fe				
	CuO	0–40 μg/cm^2^	-	Human lung epithelial cells (A549)	Mitochondria-dependent cellular apoptosis associated with DNA damage [88].
	SPION	200–1000 μg/mL, (24 h)	Increased cytokines, inflammation, TNF-α [89].	Human lung epithelial cells (A549)	Activation of JNK, stimulation of tumor necrosis factor-alpha (TNFα), reduction of NF-kB, increased ROS [90].
	SWCNT	10–100 μg/mL (24 h, 48 h and 72 h)	Dose- and time-dependent decline in cell viability: up to 50% decrease at maximum dosage after 72 h. Oxidative stress was exhibited as a mechanism of cytotoxicity [91].	Human lung epithelial cells (A549)	low acute toxicity was confirmed with the in vivo model by dispersion of SWCNTs in serum [92].
	QD	12.5 µg, (7 days)	Increased levels of LDH and albumin [93].	Human lung adenocarcinoma cells	Mitochondria-dependent cellular apoptosis associated with decrease of cells viability [94].
Heart	AgNPs	100, 1000 and 10,000 ppm, (period of 13 weeks)	increasing cardiocyte deformity, congestion and inflammation [71].	Catla heart cell line (SICH)	Increased lipid peroxidation (LPO) level and decreased level of GSH, SOD and CAT [95].
	Iron oxide NPs	100, 200, 300 and 500 μg/mL, (period of 2 weeks)	Showed that baseline maximal oxidative capacities were proteins in the heart [96].	Cardiac microvascular endothelial cells	Induced a concentration- and time-dependent cytotoxicity with decrease of cells viability
	CNT	1–0.3 mg/Kg body weight	Blocks potassium channels. The suppressed and inhibited IK and potassium channels lead to increased heart rate [97].	Microvascular Endothelial Cells	Dose- and time-dependent increasing DNA damage [98].
	QD	-	-	Human hepatocellular carcinoma, HepG2 cells	Mitochondria-dependent cellular apoptosis associated with ROS [99].
Dermal	AgNP	50 and 100 μg/mL, (24 h)	Mitochondria-dependent cellular apoptosis related to ROS at a concentration of ≥ 50 μg/mL [100].	A431 (human skin carcinoma)	No evidence for Cellular damage up to a concentration of 6.25 g/mL. Morphological changes at concentrations between 6.25 and 50 g/mL with concomitant rise in GSH, SOD and lipid peroxidation. DNA fragmentation suggests cell death by apoptosis [101].
	TiO_2_	15 μg/cm^2^, (24 h)	Cytotoxicity was detected to be apoptosis [101].	HaCaT (keratinocyte cell line), human dermal fibroblasts, human immortalized sebaceous gland cell line (SZ95)	Cytotoxicity was observed to be affecting cellular functions such as cell proliferation, differentiation and mobility resulting in apoptosis [70].
	Fe_3_O_4_	65 nm	-	Skin tumor cells	Increases ROS, deceasing cancer cells [102].
	CNT	10 μg/mL, (72 h)	NPs increased relatively IL8 and ROS factors in animal [103].	Human Dermal Fibroblast Cells	Mitochondria-dependent cellular apoptosis associated with decrease cell viability [104].
	QD	4.6 nm core/shell diameter QD for 8 h and 24 h	Increased IL-1b, IL-6, and IL-8 [105].	Human epidermal keratinocytes (HEKs)	Increased IL-1β, IL-6, IL-8, and TNF-α factors [106].
Liver	AgNPs	10, 50, 100, 150, 200, 400 ppm for 24 h	-	Primary mouse fibroblasts, primary hepatocytes	Production of mediators of oxidative-stress. increase GSH [107].
	CdSe	62.5–1000 µg/mL, (1–8 h)	-	Primary rat hepatocytes	Evidence for cellular damage up to a concentration of 62.5 µg/mL with concomitant rise in GSH, SOD and lipid peroxidation [108].
	ZnO NPs	100, 300 and 600 mg/Kg, (7 days)	-	Human hepatocyte (L02)	Mitochondria-dependent cellular apoptosis associated with ROS, reduction of SOD, depletion of GSH, and oxidative DNA damage [91].
	Al_2_O_3_	235,245 ppm	Blood cell and melanoma macrophage accumulation, hepatocyte necrosis, vaculation and portal vein alteration [109].	-	-
	TiO_2_	5, 10, 50, 100 or 150 mg/Kg, (daily for 14 days)	NPs increased relatively IL-8 and ROS factors in animal [110].	Rat liver derived cell line (BRL 3A)	Mitochondria-dependent cellular apoptosis associated with ROS, reduction of SOD, depletion of GSH, and oxidative DNA damage [9].
	CNT	~25 μg/cm^2^	NPs increased relatively apoptosis factor in animal [111].	Human hepatoblastoma C3A cell line	Mitochondria-dependent cellular apoptosis associated with ROS, IL8, reduction of SOD, depletion of GSH, and oxidative DNA damage [112].
	QDs	1000 µg/mL, (24 h)	NPs increased relatively ROS in liver [113].	Primary rat hepatocytes	Cytotoxicity was thought to be due to the release of free cadmium ions [108].
Kidney	AuNPs	5, 10,100 ppm, (via IP injection for 7 successive days)	Increase levels of CREA, UREA, total bilirubin ALP in rats’ blood serum were examined to show a degree of kidney functionality [114].	Embryonic kidney cells (HEK293).	Toxicity was dose dependent. In a dose of 44 mg/mL for 4 h, toxicity was observed on DNA/transferrin [115].
	ZnO NPs	100, 300 and 1000 mg/Kg in 2 weeks	Significant increase in serum creatinine and blood urea nitrogen, decrease in hemoglobin, haematocrit and mean corpuscular hemoglobin concentration [116].	Human embryonic kidney (HEK293) cells	Lead to cellular morphological modifications, mitochondrial dysfunction, and cause reduction of SOD, depletion of GSH, and oxidative DNA damage [91].
	CuO NPs	A dose of 10 mg/Kg three times a week up to 19 injections	Toxicity showed with DNA fragmentation [117].	Embryonic kidney cells (HEK293)	Increased ROS, decreased cell viability [118]
	TiO_2_	1, 10, 100 µg/mL	Embryonic kidney cells	-	DNA damage and genomic toxicity [119].
	CNT	4 mg/Kg, (7 days)	Increase level of IL-8, LDH and lipid peroxidation in serum [120].	Embryonic kidney cells (HEK293)	Decreased cell viability, increase cell membrane damage, LDH release, reduced glutathione (GSH), interleukin-8 (IL-8), lipid peroxidation [121]
	QD	1.5 µmol/Kg, (1, 7, 14, and 28 days)	-	Embryonic kidney cells (HEK293)	Time-dependent decrease of mitochondrial transmembrane potential, up regulate Bcl-2 expression, alleviated apoptosis [122].
Spleen	AgNPs	30, 300 and 1000 mg/Kg doses of AgNPs (60 nm), 28 days of oral administration	Ag induces the permeability of cell membrane to potassium and sodium and interrupts the activity of Na-K-ATPase and mitochondria. Inhibition of NF-kB activity, a decrease in expression bcl-2, increase in caspase-3 expression [123,124].	-	-
	Fe_2_O_3_	0.1, 0.5 and 1.0 mg/L (9.2 × 10^−4^, 4.6 × 10^−3^ and 9.2 × 10^−3^ mM) aqueous suspensions for 60 days	Accumulated in the spleen organ and induce acute toxicity [125].	-	-
	CNT	1.5 mL; 2 mg multi-walled (MW) CNT per body weight (bw), (1, 6, 24, 48 and 144 h)	After i.p. administration, MWCNT translocate progressively in the spleen, with a peak of concentration after 48 h, and determine lymphoid hyperplasia and an increase in the number of cells which undergo apoptosis, in parallel with the enhancement of the mitosis in the white pulp and with transient alterations of oxidative stress and inflammation [126].	-	-
	QD	6000 *g* for 10 min,	Distribution in different body organs and aggregation in spleen [127,128,129]	-	-
Stomach	AgNPs	28-day repeated oral dose of AgNPs of 60 nm, 2.6 mg Ag/Kg b.w./day	Aggregation in stomach tissues [130].	-	-
	Au NPs	-	-	Gastrointestinal cancer cells	Removing tumor cells from healthy cells [131].
	CdSe	0.84 × 10^5^ µm	-	Human colon carcinoma cell line	Removing tumor cells from healthy cells [132].
	TiO_2_ NPs	1012 particles/person per day in 2 weeks	Aggregation in stomach tissues [133].	-	-
	ZnO NPs	5, 50, 300, 1000 and 2000 mg/Kg b.w	Aggregation in stomach tissues [134].	-	-
	CNT	<5 µm, 10–20 µm, (7 days)	Inflammation [135,136,137,138]	-	-
	QD	2 to 200 nmol/mL, (24 h)	NPs increased relativel ROS factors in animal [139]	-	-
Pancreas	Ag NPs	AgNPs (100 μg/mL), (24 h)	NPs increased relatively ROS factors in animal [140].	Pancreas cancer BxPC-3 Cells	Inhibition of NF-kB activity, a decrease in bcl-2, and an increase in caspase-3 and survivin expression [141].
	AuNPs	50 nm, 2.5 mg/Kg, Male Wistar diabetic with autism spectrum disorder pups, i.p. 7 day	NPs increased relatively ROS factors in animal [142]	-	-
	cobalt ferrite NPs	-	-	human pancreatic cancer cells	Accumulation in cells increasing apoptosis [143].
	ZnO NPs	0, 500, 1000, and 2000 mg/Kg/day for 14 days.	Decreased body weight, feed consumption, alterations in blood factors (HB, HCT, MCV, MCH, MCHC, and LYM) and increase in blood cells (WBCs and NEUs), and histopathological alterations in the pancreas [144].	-	-
	TiO_2_	42 days	-	Pancreatic cancer cells (PANC-1)	Tumor growth inhibition and induce cell toxicity [145].
	CNT	5, 10 and 50 μg/mL	-	Pancreatic cancer cells (PANC-1)	Hyperthermia; necrosis of malignant cells [146]
	QD	0.2 mL, (7 h)	NPs increased relatively ROS factors in animal organ [147].	-	-
Ear	AgNPs	4000 μg/mL AgNPs induced	Hearing loss with partial recovery within 7 days and increasing ROS in animal organ [148].	BALB/c 3T3 cell line	Impairment of the mitochondrial function [148].
	SPION	150 μL of 15 mg/mL, (1–4 h, 4 and 7 days)	Uptake into the CNS parenchyma [149].	-	-
	CNT	150 μL of 15 mg/mL, (1, 2, 4 h, 4 and 7 days	Accumulation in CNS parenchyma. No pathological alterations were observed [150].	-	-
	QD	1 mg/mL or 4.5 mg/mL), (24 h)	Limb abnormalities, body wall defects, neural tube defects [150].	-	-
Eye	AuNPs	2, 20 and 200 nm, 72 h	NPs increased relatively ROS factors in animal [151].	Human corneal cells	Increasing apoptosis and aberrant expression factor pigmentation, development (pax6a, pax6b, otx2, and rx1) and pigmentation (sox10) [151].
	Iron oxide	2, 20 and 200 nm, (72 h)	NPs increased relatively ROS factors in animal [151].	-	-
	Silica NPs	50, 100 and 150 nm, (48 h)	NPs increased relatively ROS in cell [152].	-	-
	CNT	Up to 750 nm every week for 9 weeks	Eye-irritation, retinal degeneration [74].	-	-
	QD	17 weeks of age, in the range of 2.7–3.6 Kg in body weight	Eye-irritation, retinal degeneration [153].	-	-

## Abbreviations

AD	Albumin Derivatized
BBB	Blood–Brain-Barrier
BMECs	Brain Microvascular Endothelial Cells
CNS	Central Nervous System
CSF	Cerebrospinal Fluid
DD	Dextran Derivatized
Ag NP	Silver Nanoparticle
DA	Dopamine
Au NP	Gold Nanoparticles
MRI	Magnetic Resonance Imaging
CNTs	Carbon nano tubes
MWCNTs	Multi-Walled Carbon Nanotubes
NPs	Nanoparticles
QDs	Quantum Dots
SWCNTs	Single-Walled Carbon Nanotubes
SPIONs	Superparamagnetic Iron Oxide Nanoparticles
USPIONs	Ultra-Small Superparamagnetic Iron Oxide Nanoparticles
